# Atlas of RNA sequencing profiles for normal human tissues

**DOI:** 10.1038/s41597-019-0043-4

**Published:** 2019-04-23

**Authors:** Maria Suntsova, Nurshat Gaifullin, Daria Allina, Alexey Reshetun, Xinmin Li, Larisa Mendeleeva, Vadim Surin, Anna Sergeeva, Pavel Spirin, Vladimir Prassolov, Alexander Morgan, Andrew Garazha, Maxim Sorokin, Anton Buzdin

**Affiliations:** 1 Shemyakin-Ovchinnikov Institute of Bioorganic Chemistry, Moscow, 117997 Russia; 2 Department of Pathology, Faculty of Medicine, Lomonosov Moscow State University, Moscow, 119991 Russia; 3 Pathology Department, Morozov Children’s City Hospital, 4th Dobryninsky Lane 1/9, Moscow, 119049 Russia; 4 Bureau of Forensic Medicine, Moscow, Russia; 5 Department of Pathology and Laboratory Medicine, University of California Los Angeles, Los Angeles, CA 90095 USA; 6 National Research Center for Hematology, Novy Zykovsky proezd, 4, Moscow, 125167 Russia; 7 Engelhardt Institute of Molecular Biology, Russian Academy of Sciences, Vavilova Street, 32, Moscow, 119991 Russia; 8 Khosla Ventures, Menlo Park, CA USA; 9 Omicsway Corp., 340S Lemon Ave, 6040, Walnut, 91789 CA USA; 10 Oncobox ltd., Moscow, 121205 Russia; 11 I.M. Sechenov First Moscow State Medical University, Moscow, 119991 Russia

**Keywords:** Genetics research, Genetic databases, RNA sequencing, Gene expression analysis

## Abstract

Comprehensive analysis of molecular pathology requires a collection of reference samples representing normal tissues from healthy donors. For the available limited collections of normal tissues from postmortal donors, there is a problem of data incompatibility, as different datasets generated using different experimental platforms often cannot be merged in a single panel. Here, we constructed and deposited the gene expression database of normal human tissues based on uniformly screened original sequencing data. In total, 142 solid tissue samples representing 20 organs were taken from post-mortal human healthy donors of different age killed in road accidents no later than 36 hours after death. Blood samples were taken from 17 healthy volunteers. We then compared them with the 758 transcriptomic profiles taken from the other databases. We found that overall 463 biosamples showed tissue-specific rather than platform- or database-specific clustering and could be aggregated in a single database termed *Oncobox Atlas of Normal Tissue Expression (ANTE)*. Our data will be useful to all those working with the analysis of human gene expression.

## Abstract (summary)

**Table:** 

Design Type(s)	gene expression analysis objective • data integration objective • organism part comparison design • transcription profiling design
Measurement Type(s)	transcription profiling assay
Technology Type(s)	RNA sequencing
Factor Type(s)	sex • age • organism subdivision
Sample Characteristic(s)	Homo sapiens • kidney • Kidney • Colon • Liver • brain • Lung • endometrium • Ovary • prostate gland • Esophagus • Stomach • Mammary gland • Thyroid gland • Pancreas • Tonsil • skeletal muscle tissue • small intestine • Adrenal gland • urinary bladder • skin of body • uterus • uterine cervix • Small intestine • tonsil • blood • bone marrow • esophagus • liver

Machine-accessible metadata file describing the reported data (ISA-Tab format)

## Background & Summary

High throughput gene expression (transcriptomic) analyses can be used in every aspect of biomedicine^1–3^, including fundamental research^4–6^ and molecular diagnostics^7^. Growing amount of transcriptomic data is deposited in the special public repositories like Gene Expression Omnibus, GEO^8^ and Array-Express^9^ which have already accumulated over two million individual transcriptomic profiles obtained in over 100,000 series of experiments^10^. The data cover a wide spectrum of specific human physiological conditions, including most of known diseases and developmental features^8,9,11,12^.

The enclosed transcriptional profiles were obtained using different experimental platforms of either microarray hybridization or next-generation sequencing (NGS) of mRNA. However, the gene expression may be poorly comparable across the different platforms because of use of different equipment and reagents^13–17^. The NGS methods have become the standard in the field of gene expression studies because of higher reproducibility and lower platform bias^18^.

Comprehensive studies of molecular pathology or tissue specific patterns require a collection of reference samples^19,20^. Ideally, they should represent normal tissues from healthy donors, profiled in a single series of experiments using the same equipment and reagents. Nowadays, there is a shortage of such gene expression data. The largest published dataset, GTEx^21^ (11,688 samples), lacks publicly available data on the donors’ age, and this puts limits on data analysis and makes impossible age-matched studies, which are crucial for many practical applications and aging research. For that reason, we didn’t include GTEx data in this study.

The other relevant databases of significant size do include the information on the donors’ age: TCGA^22^ (625 samples), ENCODE^23^ polyA RNA-seq (38 samples), and ENCODE total RNA-seq (89 samples). However, they lack one or several of the previously mentioned features. For example, in The Cancer Genome Atlas (TCGA) project database, specimens of histologically normal tissue adjacent to surgically removed tumors^24^ are considered normal. However, these tissues may not be completely normal due to numerous effects tumors may have on the neighboring cells, including biased growth factors and cytokine balances^25^, pathological inflammation^26^, and altered vascularization^27^. The ENCODE polyA RNA-seq and ENCODE total RNA-seq datasets were generated based on the normal tissues subjected to NGS using different library preparation methods. They have only 1–4 samples profiled per tissue type (both male and female donors included) and most of the cases can not form a statistically significant reference group.

During our study we have designed and assembled the gene expression database of normal human tissues based on uniformly obtained original sequencing data using Illumina HiSeq-3000 engine. A total of 142 solid tissue samples representing 20 organs were taken from post-mortem human healthy donors, who had died in road accidents, no later than 36 hours after death. Blood samples were taken from 17 healthy volunteers. The materials, that had been being collected since 2012, were stored until gene expression profiles were obtained in one series of experiments using the same reagents and protocols. The gene expression profiles were then submitted to Gene Expression Omnibus under accession id GSE120795^28^. We also compared our data for consistency with the transcriptomic profiles taken from databases The Cancer Genome Atlas (TCGA), ENCODE polyA RNA-seq, and ENCODE- total RNA-seq. We found that 463 biosamples showed tissue-specific rather than platform- or database-specific clustering. These have beeen aggregated in a single database named *Oncobox Atlas of Normal Tissue Expression (ANTE)* (can be found on Figshare^29^ in the file “ANTE overview”), including 11 sex-matched statistically significant tissue groups. Our data will be useful to all those working with the analysis of human gene expression.

## Methods

### Biosamples

In the period from March 2012 to May 2018, we obtained 142 solid tissue samples from 20 human organs at the Department of Pathology at the Faculty of Medicine, Moscow State University, Russia, from autopsies taken from 23 non-related adult healthy donors killed in road accidents, no later than 36 hours after death. We also collected normal blood samples from 17 healthy volunteers. For each biosample an informed written consent to participate in the study was obtained from the patient’s legal representative. The consent procedure and the design of the study were approved by the ethical committees of the Faculty of Medicine, Moscow State University, and of the National Research Center for Hematology. The biosamples were evaluated by a pathologist to confirm the tissue origin of every specimen prior to analyses.

Overall, blood and solid tissues were taken from 8 male and 9 female and 14 male and 9 female donors, respectively. The mean age was 33.47 years old (range 23–75 y.o.) and 37.39 y.o. (12–54 y.o.), respectively. The full list of the tissues and biosamples obtained is given in Online-only Table 1.

### Preparation of libraries and RNA sequencing

#### RNA extraction

Solid tissue samples were either immediately stabilized in RNAlater (Qiagen, Germany) and then stored at −70 °C or fixed in formalin and embedded in paraffin blocks. RNA extraction was performed immediately before the preparation of sequencing libraries using QIAGEN RNeasy Kit (Qiagen) or Direct-zol™ RNA MiniPrep (Zymo Research) with TRI Reagent (MRC) for tissues in RNAlater and the RecoverAll™ Total Nucleic Acid Isolation Kit for FFPE (Invitrigen), following the manufacturer’s protocol. In cases of whole blood normal samples, mononuclear cells were extracted shortly after peripheral blood collection. Alternatively, a fraction of CD138 + cells was isolated from bone marrow with Ficoll Paque Plus (Sigma) followed by enrichment using CD138 MicroBeads (Miltenyi Biotec) and MS Columns (Miltenyi Biotec). Cells were counted by Scepter™ 2.0 Handheld Automated Cell Counter (Merck Millipore) and immediately subjected to RNA extraction. For RNA extraction, cells were resuspended in TRI Reagent (MRC) and then Direct-zol™ RNA MiniPrep (Zymo Research) was used for the RNA extraction. RNA was quantified using Nanodrop (Thermo Fisher Scientific), ethanol-precipitated, and stored in liquid nitrogen until sequencing.

#### Library preparation

RNA Integrity Number (RIN) was measured using Agilent 2100 bioanalyzer. Agilent RNA 6000 Nano or Qubit RNA Assay Kits were used to measure RNA concentration. KAPA RNA Hyper with RiboErase (KAPA Biosystem) Kit was used for further depletion of ribosomal RNA and library preparation. Different adaptors were used for multiplexing samples in one sequencing run. Library concentrations and quality were measured using Qubit ds DNA HS Assay kit (Life Technologies) and Agilent Tapestation (Agilent). RNA sequencing was performed using Illumina HiSeq 3000 equipment for single end sequencing, 50 bp read length, for approximately 30 million raw reads per sample. Data quality check was conducted using Illumina SAV. De-multiplexing was performed using Illumina Bcl2fastq2 v 2.17 software.

#### Processing of RNA sequencing data

RNA sequencing FASTQ files were processed with STAR aligner^30^ in ‘GeneCounts’ mode with the Ensembl human transcriptome annotation (Build version GRCh38 and transcript annotation GRCh38.89). Ensembl gene IDs were converted to HGNC gene symbols using Complete HGNC dataset (https://www.genenames.org, database version of July 13, 2017. In total, expression levels were established for 36596 annotated genes with corresponding HGNC identifiers. Additional QC metrics for obtained data were generated using NCBI MAGIC software^18,31,32^. All metrics and detailed protocol for each sample can be found on Figshare^29^ in the file “QC and additional meta-information”.

#### Data clustering

‘1’ was added to all raw gene counts prior to cluster analyses, to avoid zero expression values, as described by Dillies *et al*.^33^, the gene expression data were merged into single datasets and quantile normalized^34^. Hierarchical clustering was performed using R ward.D2 method. The dendrogram was visualized using custom R script.

## Data Records

Gene expression profiles were deposited at the Gene Expression Omnibus database (GEO) under accession number GSE120795^28^. The data is provided as a matrix of raw counts as generated by STAR. The raw data can be downloaded from the Sequence Read Archive (SRA^35^). The mapping statistics for corresponding dataset can be found on Figshare^29^ in the file “QC and additional meta information”. The available RNA sequencing profiles for normal and cancer tissues matched normal samples were extracted from the websites of projects TCGA and ENCODE (portal.gdc.cancer.gov and www.encodeproject.org, respectively). Combined meta information for compatible gene expression profiles from different databases can be found on Figshare^29^ in the file “ANTE overview”.

## Technical Validation

### RNA sequencing data quality control and consistency tests

**Fig. 1:**
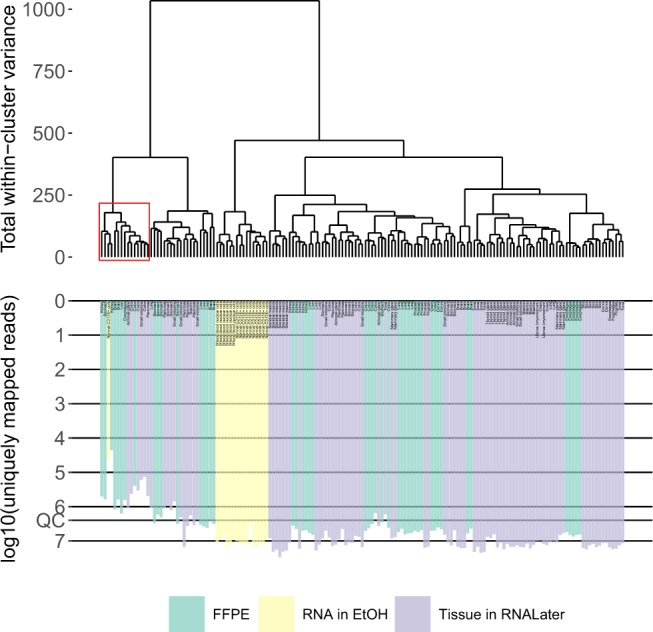
The hierarchical clustering dendrogram of all experimental RNA sequencing profiles of human tissues. Gene expression data were used to calculate Euclidian distances between the samples. Color indicates the sample preparation method (tissue in FFPE, RNA in ethanol, tissue in RNAlater). The lower scale indicates the number of uniquely mapped reads. QC denotes the quality control threshold of 2.5 million uniquely mapped reads.

To assess if the obtained gene expression profiles are in correlation with the biological nature of the biosamples tested and identify samples that might be of low quality, we performed cluster analysis of all the RNA sequencing data we had obtained (Fig. 1). The color on the dendrogram indicates technical type of biosamples: *FFPE* or *tissue in RNAlater* for solid tissue, and *RNA in ethanol* for blood samples.

The FFPE and RNAlater samples formed several mixed clusters on the dendrogram, which suggests that the clustering was independent of the sample preparation technique for solid tissues. The blood samples formed a clearly distinct cluster on the left side of the dendrogram, which is in line with the histological status of this tissue (Fig. 1), which stand apart among other samples.

**Fig. 2:**
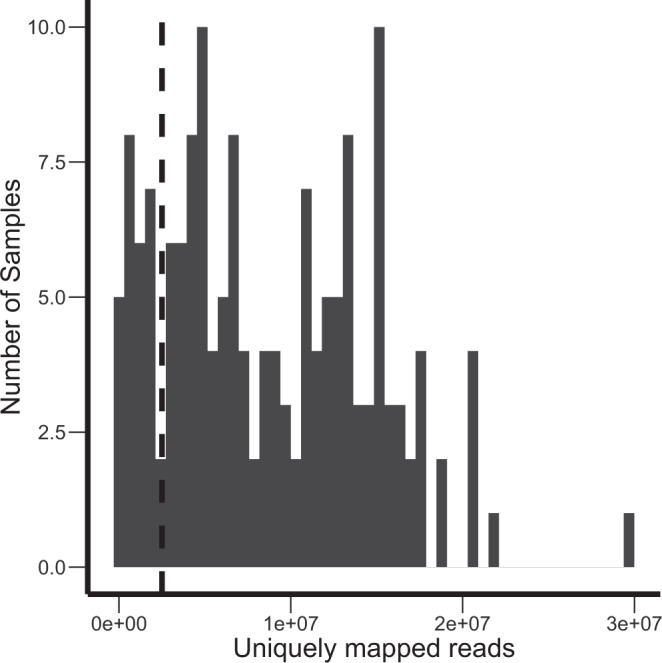
The distribution of the experimental RNA sequencing profiles with respect to the number of uniquely mapped reads. The vertical dashed line indicates the QC threshold of 2.5 million uniquely mapped reads per sample.

**Fig. 3:**
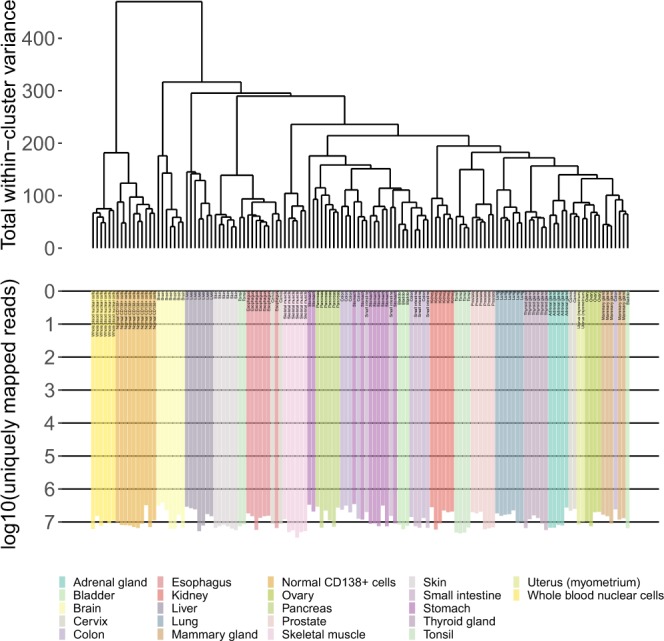
The hierarchical clustering dendrogram of QC-passed experimental RNA sequencing profiles of human tissues. Gene expression data were used to calculate Euclidian distances between the samples. The color markers indicate the tissue types. The lower scale indicates the number of uniquely mapped reads. ‘QC’ denotes the quality control threshold of 2.5 million uniquely mapped reads.

However, we then observed a mixed cluster next to the one formed by the blood samples, which included both blood and solid tissue specimens (Fig. 1, red), and such clustering did not correspond to physiological origin of biosamples. We noticed that that cluster was formed exclusively by the samples with relatively low (less than 2.5 million) number of uniquely mapped sequencing reads (Fig. 1, scale) and hypothesized that this may represent a deviation which arose due to insufficiency of data. We analyzed contents of the samples with respect to the number of uniquely mapped reads (Fig. 2) and found that the samples with lowest number of reads indeed formed a distinct cluster with the upper threshold of ~2.5 million reads uniquely mapped to HGNC genes (Fig. 2). We, therefore, used this threshold, which enabled us to separate effectively solid tumors from hematological ones, as an indicator for quality control (QC) of the sequenced gene expression profiles. 132 samples out of a total of 159 passed the QC threshold (Table 1). After the QC filter was applied RNA sequencing profiles became clustered in the hierarchical way following the histological origin of the tissues (Fig. 3).

**Table 1:** Human tissue samples included in the RNA sequencing assay.

Tissue	# of samples	# of samples passed QC
Adrenal gland	6	5
Bladder	5	4
Brain	9	7
Cervix	4	4
Colon	12	7
Esophagus	8	7
Kidney	8	6
Liver	8	7
Lung	8	7
Mammary gland	5	5
Normal CD138+cells	11	10
Ovary	4	4
Pancreas	8	6
Prostate	6	6
Skeletal muscle	6	6
Skin	6	6
Small intestine	9	5
Stomach	15	10
Thyroid gland	6	6
Tonsil	7	6
Uterus (myometrium)	2	2
Whole blood nuclear cells	6	6
**Total**	**159**	**132**

The established threshold effectively marked samples with low quality values of other QC metrics, e.g. proportion of genomic counts, high rate of mismatches, number of reads spanning splice junction, high percentage of ribosomal counts. Full list of mapping statistics generated by STAR aligner and additional QC metrics generated by NCBI MAGIC is available on Figshare^29^ in the file “QC and additional meta information”.

**Fig. 4:**
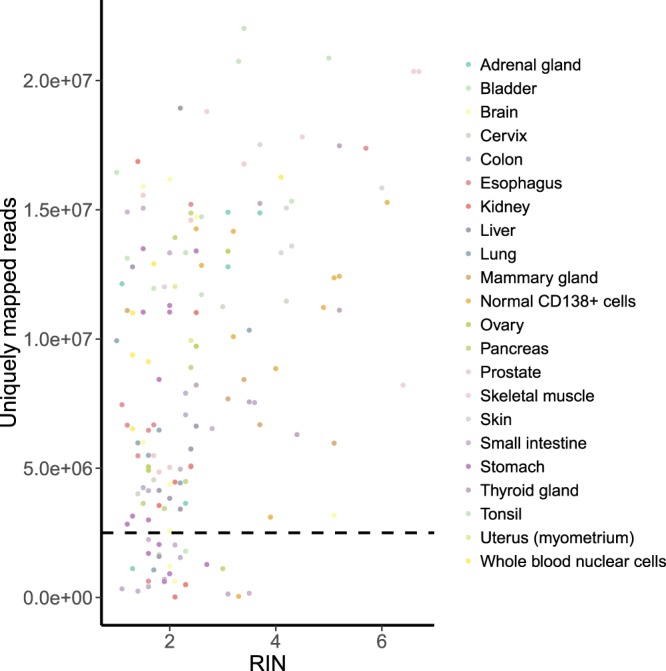
RIN vs number of uniquely mapped reads per sample. Spearman’s rho = 0.344 (p-value = 9.687e-06). The horizontal dashed line indicates the QC threshold of 2.5 mln uniquely mapped reads.
(ii) RNA concentration. We found no correlation between RNA concentration and number of uniquely mapped reads (Fig. 5). Therefore, low RNA concentration was not a significant indicator for quality assessment. In most of the cases, low RNA concentration still allowed the sample to pass the QC.

We then looked for a correlation between whether a biosample passes the QC and its internal characteristics. The following parameters were investigated:

(i) RIN, that shows the level of RNA degradation (lower RIN points to more degraded RNA). All samples with high RIN (RIN > 4) passed the QC (Fig. 4). However, low RIN turned out not to be an informative marker of the insufficient number of reads, and most of the samples with 1 < RIN < 2 passed the QC as well.

**Fig. 5:**
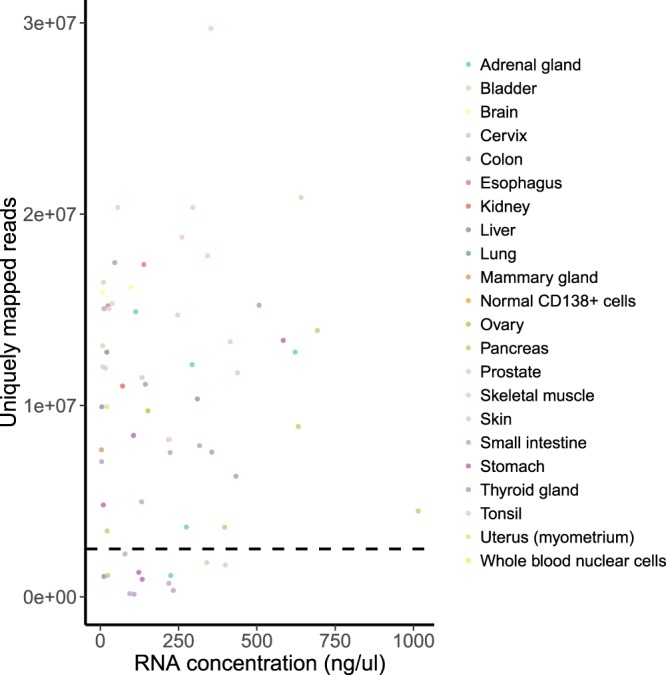
RNA concentration vs number of uniquely mapped reads per sample. Spearman’s rho = 0.03 (p-value = 0.8). The horizontal dashed line indicates the QC threshold of 2.5 mln uniquely mapped reads.

Only the samples that had more reads than the QC threshold were analyzed in further comparisons.

**Fig. 6:**
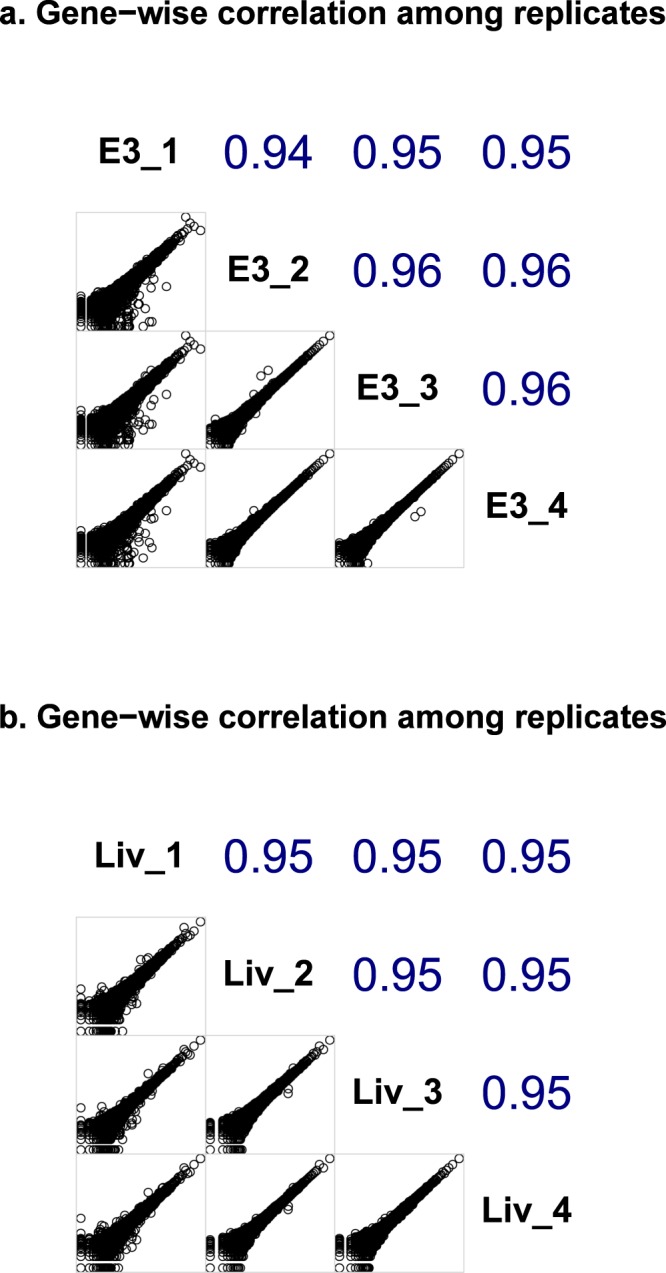
The correlation plots for four gene expression profiles in replicate RNA sequencing experiments. (**a**) Comparison for the esophagus, E_3 tissue biosample. (**b**) Comparison for the liver, ID 15_6 tissue biosample. Upper part of the diagonally split matrix shows correlation coefficients (Spearman’s rho). Bottom diagonal shows pairwise plots for gene expression values in logarithmic scale for every pair of replicates under comparison.

Finally, we assessed the reproducibility of the RNA sequencing output data for the characterization of normal human tissues. We performed RNA sequencing of four different fragments of each of the following tissue specimens: two human liver (ID 16_5) and esophagus (ID E_3). The tissue fragments were blinded and sent for sequencing separately. In each case, one of quadruplicates was sequenced and mapped in a separate batch. In both cases we observed high pairwise correlation coefficients between quantile normalized gene expression values (Spearman’s rho ≥ 0.94 in all cases) (Fig. 6a,b). We concluded that the obtained gene expression profiles are highly reproducible among the replicates for materials taken from different portions of the same biosample.

### Compatibility with other gene expression datasets

**Fig. 7:**
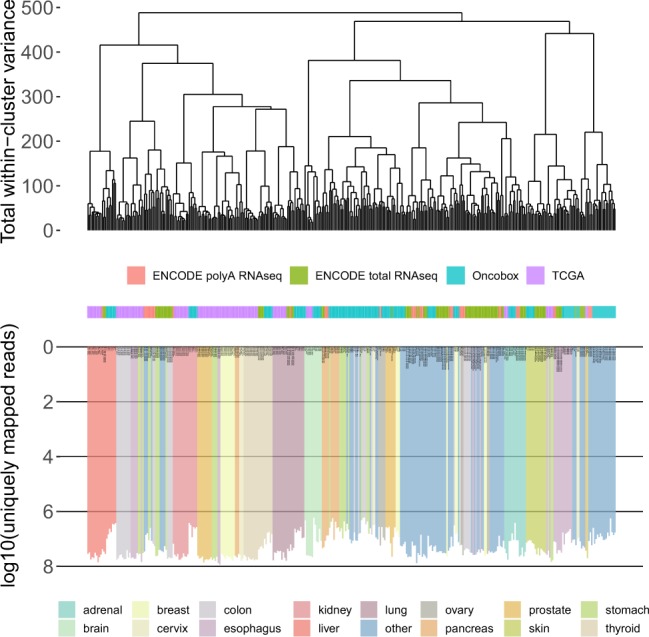
The dendrogram of normal samples from Oncobox (no prefix in sample names), TCGA database (“TCGA_” prefix in samples names) and ENCODE (“ENCODE_” prefix in samples names). For the TCGA data 10 random samples per tissue type were selected for visualization, in cases when more norms were available. Euclidian distance between the samples was measured using gene expression data. The dendrogram was built using R ward.D2 method. The color markers indicate the tissue type. The lower scales indicate the number of uniquely mapped reads.

After that we checked if our gene expression data are compatible with previously published transcriptomic datasets. The largest published datasets of RNA sequencing data for normal tissues were TCGA database^22^ of ‘normal’ tissues adjacent to tumors (625 samples, see the file “TCGA gene expression data” on Figshare^29^), ENCODE^23^ database, poly(A) priming library preparation (38 samples, see the file “ENCODE polyA RNA-seq gene expression data” on Figshare^29^), and ENCODE^23^ database, random priming library preparation (see the file “ENCODE total RNA-seq gene expression data” on Figshare^29^). All the above mentioned gene expression profiles, both obtained in our experiments and published earlier, were merged and quantile normalized^34^. We then performed cluster analysis of these data and found that both ENCODE databases showed clustering similar to that of the current experimental database, with clear tissue specific, rather than database specific, clustering patterns (Fig. 7). Contrariwise, most of the samples from the TCGA database clustered in a database specific way (Fig. 7), probably due to cancer-associated pathological changes in the respective tissue specimens. However, the TCGA samples of lung, liver, kidney, thyroid gland, adrenal gland, and brain clustered according to the tissue they represent along with the samples from other datasets (Fig. 7). We then assembled all the biosamples in all four datasets, that clustered in the tissue-specific way, in a single database named *Oncobox Atlas of Normal Tissue Expression (ANTE)*. The database overview is presented in the Table 2, the detailed information can be found on Figshare^29^ in the file “ANTE overview”. In total, the *ANTE* database contains 463 transcriptomic profiles for 11 tissue types. All the types of tissue are represented by seven samples or more, with the mean representation of 42 samples per tissue.

**Table 2:** Overview of *Oncobox Atlas of Normal Tissue Expression (ANTE)* database.

Tissue	Number of samples
Adrenal gland	12
Brain	12
Esophagus	13
Kidney	136
Liver	60
Lung	123
Ovary	8
Pancreas	9
Prostate	7
Skin	14
Thyroid gland	69

## Usage Notes

We have assembled the gene expression database of normal human tissues termed *Oncobox Atlas of Normal Tissue Expression (ANTE)*. The database includes 67 original experimental and 396 previously published gene expression profiles for 11 normal human tissues. The experimental profiles that are published here for the first time can be considered a golden standard data from the histopathological point of view as they had been obtained for the post-mortem human healthy donors, killed in road accidents, no later than 36 hours after death. Blood samples were taken from 17 healthy volunteers. The ANTE database provides unparalleled source of normal human tissues for transcriptomic research. The data in the database are in a machine-readable format and annotated by the available patients’ data which include tissue type, sex, and age of a donor. Besides comparisons of individual gene expressions, e.g. of pathological v normal human tissues, this collection of molecular data could be beneficial for a more complex level of data analysis such as molecular pathway activation scoring^36,37^ and bioinformatic ranking of drugs^38^. Additionally, the database can be used to revise lists of tissue specific gene expression biomarkers and housekeeping genes.

### ISA-Tab metadata file

Download metadata file

## Data Availability

R code for building dendrograms with bar plots is freely available on Gitlab at: https://gitlab.com/oncobox/watermelon_multisection/blob/master/utils/gallow_plot.R.

## Online-only Table

**Online-only Table 1:** Primary characteristics of tissue biosamples

Donor ID	Sex	Age, y.o.	Tissues collected
FFPE-1	Female	29	Brain, Cervix, Colon, Esophagus, Ovary, Kidney, Liver, Stomach
FFPE-2	Male	27	Brain, Esophagus, Kidney, Liver, Prostate, Stomach, Colon
FFPE-3	Male	47	Brain, Colon, Esophagus, Kidney, Liver, Lung, Prostate, Stomach
FFPE-4	Male	33	Brain, Colon, Esophagus, Kidney, Liver, Lung, Prostate, Stomach
FFPE-5	Female	50	Cervix, Colon, Esophagus, Kidney, Liver, Lung, Ovary, Brain, Stomach
FFPE-6	Female	24	Liver, Brain, Colon, Esophagus, Kidney, Stomach
FFPE-7	Male	39	Lung
FFPE-8	Male	41	Lung
Later-1	Male	36	Adrenal gland, Colon, Pancreas, Skeletal muscle, Small intestine, Stomach, Thyroid gland, Tonsil
Later-10	Female	44	Mammary gland
Later-11	Female	51	Mammary gland
Later-12	Female	42	Adrenal gland, Bladder, Brain, Cervix, Colon, Esophagus, Lung, Mammary gland, Ovary, Pancreas, Skeletal muscle, Skin, Small intestine, Stomach, Thyroid gland, Tonsil, Uterus (myometrium)
Later-13	Male	16	Adrenal gland, Brain, Colon, Pancreas, Prostate, Skin, Small intestine, Stomach, Tonsil
Later-14	Male	29	Prostate, Skin, Small intestine, Stomach
Later-15	Male	49	Prostate, Skin, Small intestine, Stomach
Later-16	Female	51	Cervix, Kidney, Liver, Mammary gland, Ovary, Pancreas, Skin, Small intestine, Stomach, Tonsil, Uterus (myometrium)
Later-2	Male	20	Adrenal gland, Colon, Lung, Pancreas, Skeletal muscle, Small intestine, Stomach, Thyroid gland
Later-3	Male	45	Adrenal gland, Bladder, Colon, Pancreas, Skeletal muscle, Small intestine, Stomach, Thyroid gland, Tonsil
Later-4	Male	42	Bladder, Pancreas, Skeletal muscle, Thyroid gland, Tonsil
Later-5	Male	44	Bladder
Later-6	Male	54	Bladder
Later-8	Female	12	Adrenal gland, Brain, Colon, Esophagus, Kidney, Liver, Lung, Pancreas, Skeletal muscle, Skin, Small intestine, Stomach, Thyroid gland, Tonsil
Later-9	Female	35	Mammary gland
WB1	Male	25	Peripheral blood mononuclear cells
WB2	Male	75	Peripheral blood mononuclear cells
WB5	Female	24	Peripheral blood mononuclear cells
WB6	Male	36	Peripheral blood mononuclear cells
WB7	Female	23	Peripheral blood mononuclear cells
WB8	Female	31	Peripheral blood mononuclear cells
D1	Female	25	Normal CD138 + cells
D2	Female	28	Normal CD138 + cells
D3	Male	33	Normal CD138 + cells
D4	Male	24	Normal CD138 + cells
D5	Female	36	Normal CD138 + cells
D6	Female	41	Normal CD138 + cells
D7	Female	30	Normal CD138 + cells
D8	Male	40	Normal CD138 + cells
D9	Male	38	Normal CD138 + cells
D10	Male	27	Normal CD138 + cells
D11	Female	33	Normal CD138 + cells
